# Centrifugation Removes a Population of Large Vesicles, or “Macroparticles,” Intermediate in Size to RBCs and Microvesicles

**DOI:** 10.3390/ijms22031243

**Published:** 2021-01-27

**Authors:** Michael C. Larson, Neil Hogg, Cheryl A. Hillery

**Affiliations:** 1 Banner University Medical Center-Tucson, University of Arizona, Tucson, AZ 85724, USA; 2 Department of Biophysics, Medical College of Wisconsin, Milwaukee, WI 53226, USA; nhogg@mcw.edu; 3 Department of Pediatrics, UPMC Children’s Hospital of Pittsburgh, Pittsburgh, PA 15224, USA; cheryl.hillery@chp.edu

**Keywords:** blood, cell membrane microparticles, circulating cell-derived microparticles, cytoplasmic vesicles, erythrocytes, flow cytometry, RBCs, centrifugation

## Abstract

Microparticles or microvesicles (MPs/MVs) are sub-cellular vesicles with a growing number of known biological functions. Microvesicles from a variety of parent cells within the vascular system increase in numerous pathological states. Red blood cell-derived MVs (RMVs) are relatively less studied than other types of circulating MVs despite red blood cells (RBCs) being the most abundant intravascular cell. This may be in part due the echoes of past misconceptions that RBCs were merely floating anucleate bags of hemoglobin rather than dynamic and responsive cells. The initial aim of this study was to maximize the concentration of RMVs derived from various blood or blood products by focusing on the optimal isolation conditions without creating more MVs from artificial manipulation. We found that allowing RBCs to sediment overnight resulted in a continuum in size of RBC membrane-containing fragments or vesicles extending beyond the 1 µm size limit suggested by many as the maximal size of an MV. Additionally, dilution and centrifugation factors were studied that altered the resultant MV population concentration. The heterogeneous size of RMVs was confirmed in mice models of hemolytic anemia. This methodological finding establishes a new paradigm in that it blurs the line between RBC, fragment, and RMV as well as suggests that the concentration of circulating RMVs may be widely underestimated given that centrifugation removes the majority of such RBC-derived membrane-containing particles.

## 1. Introduction

Microparticles or microvesicles (MPs/MVs) are small membrane vesicles shed from cells with the number and cargo varying depending on the stimuli to the parent cell [1,2,3]. Red blood cell (RBC)-derived MVs (RMVs) are formed constitutively [4,5] and in greater numbers following oxidative damage [6], hemolysis, or even ischemic injury [3,7,8]. Red blood cell-derived MVs carry biologically active macromolecules [9,10], such as exposed phosphatidylserine (PS) and phosphatidylethanolamine [11], which are critical in coagulation [12]. These phospholipids are also important in phagocytic clearance of MVs and dead cells [13,14,15] and immunomodulation [16,17]. Red blood cell-derived MV membrane proteins also contribute to inflammation [18] and coagulation [19,20]. Internally, RMVs also harbor hemoglobin, which scavenges nitric oxide [21]. Red blood cell-derived MVs have been suggested as mediators of complications of transfusion [10,21,22,23,24] and hemolytic anemia [25,26]. MPs/MVs are typically defined as particles with a diameter of 0.1–1 µm that expose phosphatidylserine, binding annexin V. Defining a standardized method by which platelet-derived MVs (PMVs) are isolated and counted has only been addressed within the past decade [27,28,29]. However, these definitions and guidelines for PMVs may not be broadly applicable. While PMVs are reportedly the most abundant type of MV circulating in healthy humans, this may differ in individuals with hemolytic anemia, where RMVs circulate in comparable concentrations [20,25]. Additionally, a growing number of reports show RBCs/RBC-derived “fragments” or MVs are critical in thrombosis [18,19,30]. While there are numerous studies addressing and comparing MV isolation procedures focused on endothelial MVs and PMVs [9,31,32,33,34], it is not clear which method is ideal for RMVs, as their parent cells (RBCs) sediment more readily than platelets or other cells. By establishing standardized protocols, cross-institution comparisons may be possible for RMVs as well as PMVs, enabling diagnoses and prognoses based on MVs [27,33].

The initial objective of this study was to understand the effects of centrifugation on RMV quantification, as there are limited reports of RMV quantification as a function of centrifugation speed [9]. Surprisingly, we discovered erythrocyte-derived debris varying in size from roughly 3 µm to sub-micron in diameter in aged donor blood that was removed with cells at low centrifugation speeds (100 g). Blood in hemolytic anemia mouse models also showed vesicles with a large variance in size. This provides evidence of a continuum of RBC-derived particle sizes, suggesting that that the arbitrary 1 µm maximal diameter cutoff for what is generally considered a “microparticle” may not apply to bilayer-encapsulated (i.e., vesicular) debris resulting from RBC hemolysis. In addition, this challenges the distinction between “RBC fragments” [30] and smaller “RBC vesicles” [7].

## 2. Results

### 2.1. Gentle Sedimentation Revealed Vesicles Larger than 1 µm in Diameter

**Figure 1:**
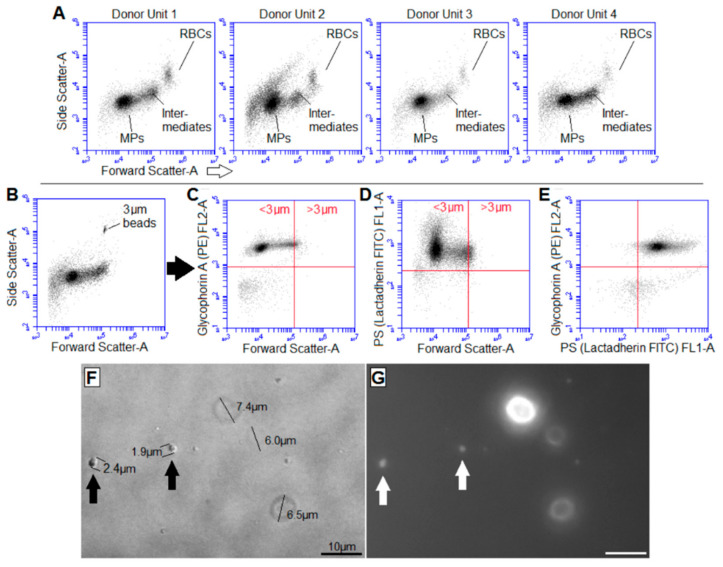
Gentle sedimentation revealed vesicles larger than 1 µm in diameter. (**A**) Representative flow cytometry light scatter (forward scatter-area, or size, and side scatter-area, or internal complexity) dot plots of RBC-poor supernatant isolated by allowing newly-outdated (43–46 day old) donor blood to stand overnight at 4 °C, (**B**) with 3 µm beads added for size comparison. Staining with (**C**) anti-glycophorin-A antibodies and (**D**) lactadherin (which binds phosphatidylserine, PS) labeled (**E**) 97.5% ± 1.1% of events in 11 expired units’ RBC-poor supernatant. (**F**) Representative bright-field image of cells and vesicles in RBC-poor supernatant and (**G**) corresponding glycophorin-A fluorescent image showing relative similar fluorescence signal from the “macroparticles” of intact RBCs; arrows highlight vesicles larger than 1 µm (“macroparticles”). Bar = 10 µm.

Flow cytometry was used to quantify RMVs in newly-outdated (43–46 day old) RBC units (Figure 1) as reported previously [11,35]. The RBC-poor supernatant was isolated by allowing RBCs to sediment by gravity overnight, as centrifugation has been shown to decrease resulting MV concentrations [9]. Platelet Endothelial Cell Adhesion Molecule-1 (PECAM-1) is a well-established surface marker of platelet and endothelial cell origin. PECAM-1 staining of outdated RBC units was not done in these experiments, as we showed previously the paucity of PECAM-1-derived vesicles in leukoreduced RBC units [11,36].

Figure 1A shows representative forward scatter (representing roughly the size for objects substantially larger than the interrogating light wavelength) and side scatter (corresponding to the internal complexity) dot plots different outdated units, displaying the heterogeneous size of debris resulting from hemolysis over the course of storage (representative 4 out of 11 outdated units). At optimal labeling conditions, only 2.5% ± 1.1% of events smaller in diameter than 6 µm were both PS and glycophorin A negative (Figure 1B–E representative of *n* = 11 outdated units) and were disregarded for subsequent analysis as either background noise or true debris devoid of a membrane surface including either PS or glycophorin A; these criteria would include both right-side out (glycophorin A+) and inside-out (glycophorin A−/PS+) vesicles. There consistently were populations of large cell-to-MV intermediates that were clearly larger than the 1 µm cut-off normally applied to MVs (which we will refer to as “macroparticles”).

Fluorescence microscopy qualitatively validated the flow cytometry data. Figure 1F is a brightfield image of RBC-poor supernatant isolated by overnight sedimentation; Figure 1G is the accompanying glycophorin A fluorescence image. Arrows highlight glycophorin A-positive “macroparticles” (i.e., vesicles larger than the 1 µm commonly accepted maximum MV diameter). Quantitative fluorescence measurements of the macroparticles were not attempted given the dependence of the fluorescence signal on the microscope focus as well as the clear inhomogeneous staining of intact RBCs. A summary of previously reported RBC vesicle sizes is shown for comparison (Table 1).

### 2.2. Size Determination of Cell-to-Microparticle Intermediate “Macroparticles”

While microscopic inspection of RBC-poor supernatant confirmed the presence of membrane vesicles larger than 1 µm in diameter, flow cytometry was used for quantification and sizing of the large vesicles. Though sub-micrometer vesicles are better characterized with side scatter (SSC) [29], the apparent diameter of the macroparticles was appropriate for using forward scatter to characterize them using the flow cytometer used in this study [36,43]; vesicles with diameters closer to the wavelength of interrogating light more appropriately are “sized” using side scatter [44].

**Figure 2:**
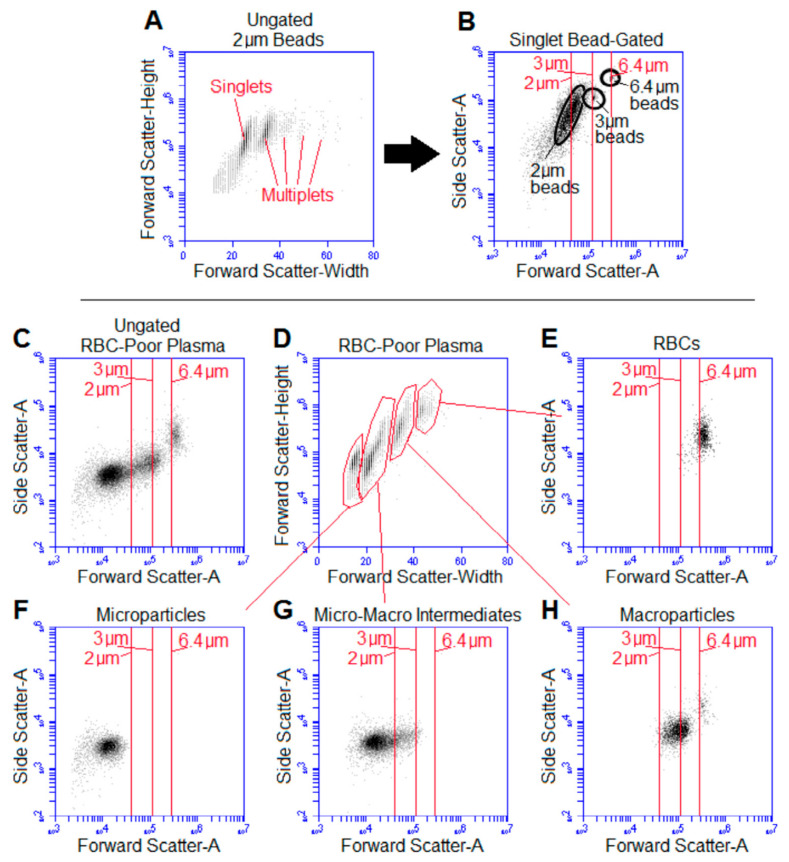
Size determination of cell-to-microparticle intermediate “macroparticles”: (**A**) Example identification of singlets in examining 2 µm beads using the forward scatter height by width; multiplets have disproportionately large width relative to height. (**B**) Light scatter of singlet beads (highlighted in the black circles) of various sizes was used to estimate the size of RBC macroparticles, with vertical lines corresponding to the forward scatter (FSC) of the singlet bead population. (**C**) Representative ungated RBC-poor supernatant with size windows shown by vertical lines corresponding to bead size based on data from (**B**). (**D**) This supernatant was examined for multiplets based on forward scatter height and width and gated to show (**E**) RBCs, (**F**) microparticles, (**G**) intermediately sized vesicles and possible microparticle doublets, and (**H**) macroparticles. Macroparticles made up 9.4% ± 9.3% of RBC-poor supernatant events smaller in diameter than 6 µm from units 1–4 days after expiration, and their diameter was 2.78 ± 0.35 µm based on their forward scatter height relative to that of the beads; *n* = 15 outdated units.

Figure 2A shows a representative dot plot of the forward scatter (FSC) height and width obtained from 2 µm beads. Clear multiplet populations were observed with disproportionate FSC width relative to height. The smallest population of beads was assumed to be singlets and used for subsequent size comparison and estimates (Figure 2B).

Figure 2C–H shows flow cytometric analysis of a representative RBC-poor plasma supernatant, gated based on its FSC-height by FSC-width (Figure 2D), showing the RBCs (Figure 2E) along with multiple vesicle populations. There was a distinct microparticle population (Figure 2F) and another population in size intermediate to the micro- and macro-particles that may include microparticle multiplets based on the FSC width being disproportionately larger than the FSC-H (Figure 2D). The vesicles we are referring to as macroparticles (Figure 2H) made up 9.4% ± 9.3% of RBC-poor supernatant events smaller in diameter than 6 µm from units 1–4 days after expiration, and their diameter was 2.78 ± 0.35 µm based on FSC-H of beads with a known size (*n* = 15 outdated units).

### 2.3. Centrifugation Removes Macroparticles

**Figure 3:**
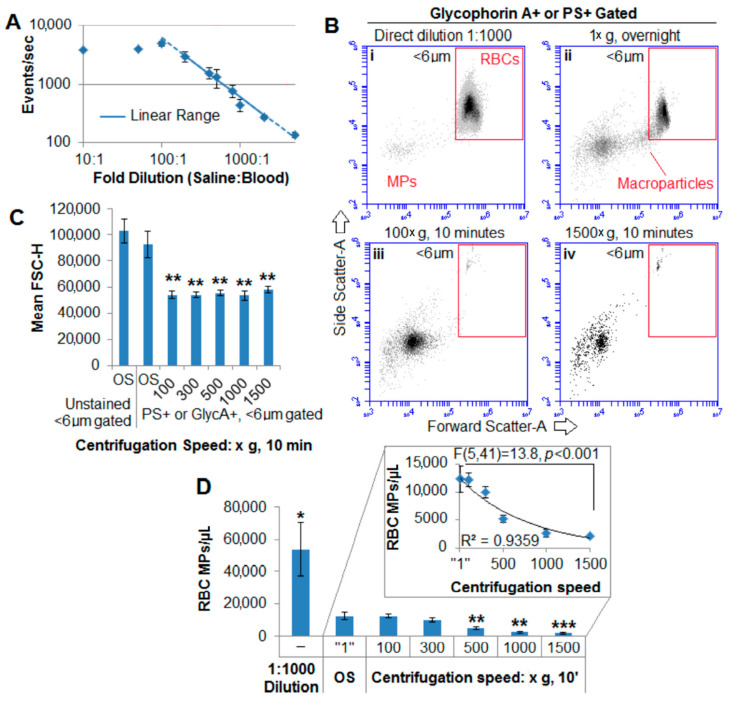
Large vesicles were removed by centrifugation: To examine the effects of centrifugation on the macroparticle population, direct dilution was carried out. (**A**) The linear range of the cytometer was first established by minimizing the variance of a linear fit of the dilution to event rate; *n* = 3 donor units to identify the midpoint of the linear range. (**B**) Representative light scatter of PS+ or glycophorin A+ events comparing the resulting detected vesicle population. (i) With direct dilution, the RBC population overwhelmed the detection of vesicles (ii) seen with overnight sedimentation. (iii) Even centrifugation at 100× *g* for 10 min removed most large vesicles, and (iv) centrifugation at 1500× *g* for 10 min resulted in removal of over 85% of vesicles compared to overnight sedimentation. (**C**) There was a significant decrease in the forward scatter height of vesicles (<6 µm) at 100× *g* or more, showing how the macroparticles are removed with low-speed centrifugation. (**D**) Compared to supernatant obtained from overnight sedimentation, there were 4.3-fold more vesicles in blood examined with the same settings used for the other supernatants. There was no significant difference in the total concentration of RBC vesicles at low speed (100 or 300× *g*), but there was a speed-dependent decrease in vesicles relative to supernatant from overnight sedimentation (inset); *n* = 8–11 (dilutions only had 8 units, whereas the different sedimentation/centrifugation numbers were performed on all 11). “1× *g*”/OS, overnight sedimentation; * *p* < 0.05; ** denotes *t*-test *p*-val. <0.001, and *** <0.0001 relative to “1× *g*”/OS.

To investigate the influence of centrifugation on the macroparticle population, centrifugation at various speeds was carried out and compared to no centrifugation (by diluting donor blood for direct measurement with flow cytometry) or overnight sedimentation (centrifugation at “1× *g*”) to produce RBC-poor supernatant. When examining blood directly without centrifugation, an appropriate dilution range was first established (Figure 3A). Figure 3B shows representative light scatter dot plots of RBC vesicles less than 6 µm in diameter by the various isolation methods. Flow cytometry of diluted donor blood resulted primarily in a signal from whole cells (Figure 3B, panel i), obstructing detection of intermediately sized vesicles/macroparticles clearly seen in Figure 3B, panel ii (overnight 1× *g* separation). Centrifugation at 100× *g* for 10 min removed the macroparticle population (Figure 3B, panel iii). This was confirmed by a significant decrease in the mean FSC of RBC-excluded events (Figure 3C). There were significantly more RBC vesicles in the directly-diluted blood compared to the overnight sediment supernatant and significantly fewer at higher centrifugation speeds (≥500× *g*) with a speed-dependent exponential decrease in the total RBC vesicles (Figure 3D, inset).

**Figure 4:**
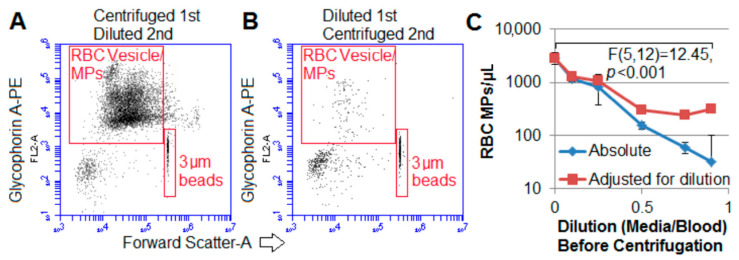
Diluting blood before centrifugation enhances microparticles or microvesicles (MV/MP) sedimentation: The timing of dilution (either before or after centrifugation) was examined. (**A**) Aliquots of donor blood were centrifuged first, their supernatant removed and diluted 1:1, and then examined. **(B**) Identical aliquots of blood were first diluted 1:1, then centrifuged, and the supernatant examined. (**A**,**B**) Representative glycophorin A and FSC dot plots of two identical aliquots centrifuged for 5 min at 50× *g* then diluted or the reverse. Immediately prior to examination with flow cytometry, 3 µm counting beads were added to doubly confirm the cytometer reported volumes, and the dot plots displayed were of identical volumes (representative of 4 donor units). (**C**) Proof-of-principle showing as the blood became more diluted, centrifugation (for 1500× *g* for 15 min) resulted in significantly fewer RMVs; *n* = 3 (1 of the aforementioned 4 samples with the most MVs/MPs performed in triplicate).

In a parallel study, the effect of dilution before or after centrifugation was investigated. Blood was either centrifuged first and then diluted in M199/Bovine Serum Albumin (BSA) media containing fluorescently labeled antibodies or diluted first and then centrifuged. Figure 4A is representative of four donor blood samples (different than the prior 15 outdated units) centrifuged at 50× *g* for 5 min and then diluted 1:1; Figure 4B was the same sample diluted first 1:1, and then centrifuged at the same conditions; 3 µm beads were added immediately before flow cytometry to confirm cytometer reported RMV concentrations. Both dot plots were of identical volumes examined by flow cytometry. Figure 4C shows that even after multiplying by the dilution factor, dilution before centrifugation resulted in significantly smaller total of RMVs in the resulting supernatant compared to undiluted and centrifuged blood supernatant. This decrease in MVs was likely due to the decreased viscosity of media compared with the blood supernatant. Solvent viscosity is important in MV centrifugation [45]. Thus, caution is required that MV-containing samples are not diluted into a less viscous media (including anticoagulant) prior to centrifugation to remove cells—otherwise, MVs will also more easily sediment as demonstrated here.

### 2.4. RBC-Derived MVs Sediment Was Relatively Faster than PECAM-1 + MVs

**Figure 5:**
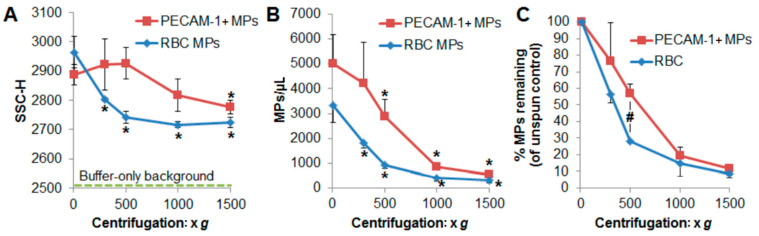
RBC MPs/RMVs sediment at lower speeds than PECAM-1 + MPs. As proof-of-principle, RBCs and platelet-rich plasma were subjected to extrusion through 0.45 µm pore size membranes to generate similarly sized MVs and, subsequently, centrifuged at different speeds before flow cytometry enumeration. (**A**) The relative size of the resulting MV populations (SSC-H) significantly decreased at all centrifugation speeds tested for RMVs, whereas there was no significant difference in the size of PECAM-1 + MVs until subjected to 1500× *g*. The green dotted line indicates the noise threshold of buffer-only liquid evaluated on the flow cytometer. (**B**) There was a significant drop in the number of RBC-MVs with any centrifugation speed; however, the decrease in PECAM-1 + MVs was not significant until 500× *g* or greater. (**C**) At 500× *g*, there were significantly fewer RMVs than PECAM-1 + MVs remaining relative to the initial unspun concentration. * Two-tailed *t*-test, *p* < 0.05 compared to unspun; # *p* = 0.010 comparing the percent remaining at 500× *g*; results are technical triplicates from a single donor.

Although RBCs are intermediate in size between leukocytes and platelets, RBCs sediment more readily than platelets and leukocytes due the fact of their density. We next examined if RBC-derived MVs would sediment quicker than platelet- and leukocyte-derived MVs. As proof-of-concept and to minimize any variance in the size of MVs or buffer effects, packed RBCs (diluted in MV-poor autologous plasma obtained by sterile filtration) and platelet-rich plasma from a single healthy donor were separately extruded through 0.45 µm filters. This resulted in MV populations with a similar side-scatter height (SSC-H) (Figure 5A) that gave a relative size approximation of small MVs of which the diameter was on the order of interrogating light wavelength [29,36,46].

Figure 5A shows the relative size of the different MV populations after extrusion and then subjected to centrifugation at various speeds. There was no difference in the SSC-H of the resulting PECAM-1 MVs with anything less than 1500× *g*. In contrast, RMVs subjected to speeds as little as 300× *g* resulted in significantly smaller MV populations. Figure 5B displays the resulting MV concentrations after centrifugation. There were significantly fewer RBC MVs even at 300× *g* centrifugation for 10 min at 4 °C; however, there was no difference in PECAM-1 + MVs compared to unspun samples at 300× *g*. When normalized to the initial unspun MV concentrations, there were significantly fewer RMVs remaining at 500× *g* compared to PECAM-1 + MVs remaining (Figure 5C). Thus, consistent with the sedimentation pattern of their parent cells; RBC-derived MVs may sediment more readily than other MV types.

### 2.5. MVs May Adhere to RBCs

As shown in Figure 3D, there was a significant discrepancy in the RMV concentration measured in dilute blood compared with supernatant isolated by 1× *g*/overnight sedimentation. To determine the fate of the “missing” RMVs, MVs were labeled with a lipophilic fluorescent dye, DiI, and the MVs washed. To control for any unbound dye, the third wash buffer was used to dilute washed RBCs. Microvesicles were then incubated at varying concentrations with RBCs before flow cytometry and fluorescence microscopy.

**Figure 6:**
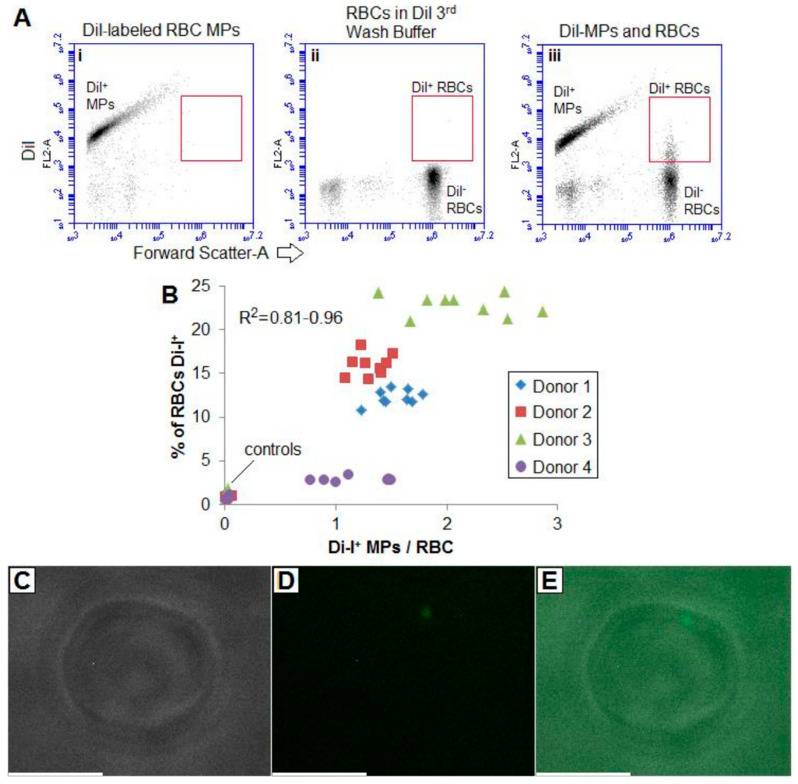
Fluorescent lipid-labeled MPs/MVs adhered to RBCs. (**A**) To further investigate the discrepancy between RMV concentrations found in direct dilutions of blood compared to RBC-poor supernatant isolated by sedimenting cells overnight, isolated MVs were labeled with DiI and washed 3x (i). The 3rd wash buffer (with any unbound dye) was used to resuspend washed RBCs (ii). When the DiI-MVs were incubated with unlabeled washed cells, there appeared to be DiI-positive RBCs (iii). (**B**) Normalized to the total cells, there was a DiI-MV concentration-dependent increase in DiI-positive cells. (**C**) Light micrograph of an RBC, with corresponding (**D**) DiI-fluorescence and (**E**) overlay show apparent MV adhered to an otherwise normal-appearing RBC. Bar = 5 µm.

Figure 6A, left panel shows the stained MVs. The wash buffer did not contain appreciable lipid dye (Figure 6A, middle panel). When stained MVs and unstained RBCs were combined, there was a population of DiI-positive RBCs that correlated to the number of DiI-positive MVs (Figure 6A, right panel); this was unrelated to the RBC concentration or cytometer flow core width, suggesting that rather than mere co-incidence on the flow cytometer, the MVs were adhering to RBCs.

Figure 6C is a brightfield image at 100× *g* of a single RBC with no apparent distinguishing features. However, the DiI-fluorescence channel (Figure 6D) showed a distinct spot that can be appreciated in the overlaid image (Figure 6E).

These results suggest that MVs may adhere to RBCs, and thus co-sediment with cells. This, in part, may explain the difference seen between RBC-poor supernatant and direct MV enumeration in uncentrifuged blood. This also highlights how even bypassing centrifugation completely by directly diluting blood and then quantifying MVs still will result in underestimates of the true concentration of vesicles within the cellular-rich biological fluid.

### 2.6. RBC-Derived Macroparticles Are Also Present in Murine Hemolytic Blood

To examine if the populations of erythrocyte macroparticles and intermediate vesicles were limited to stored blood, blood from 2 different murine models of hemolytic anemia were examined. Uncentrifuged blood from healthy mice and two different models of hemolytic anemia was examined for RBC vesicles using TER119, an antibody against murine glycophorin A, an erythrocyte lineage marker [47].

**Figure 7:**
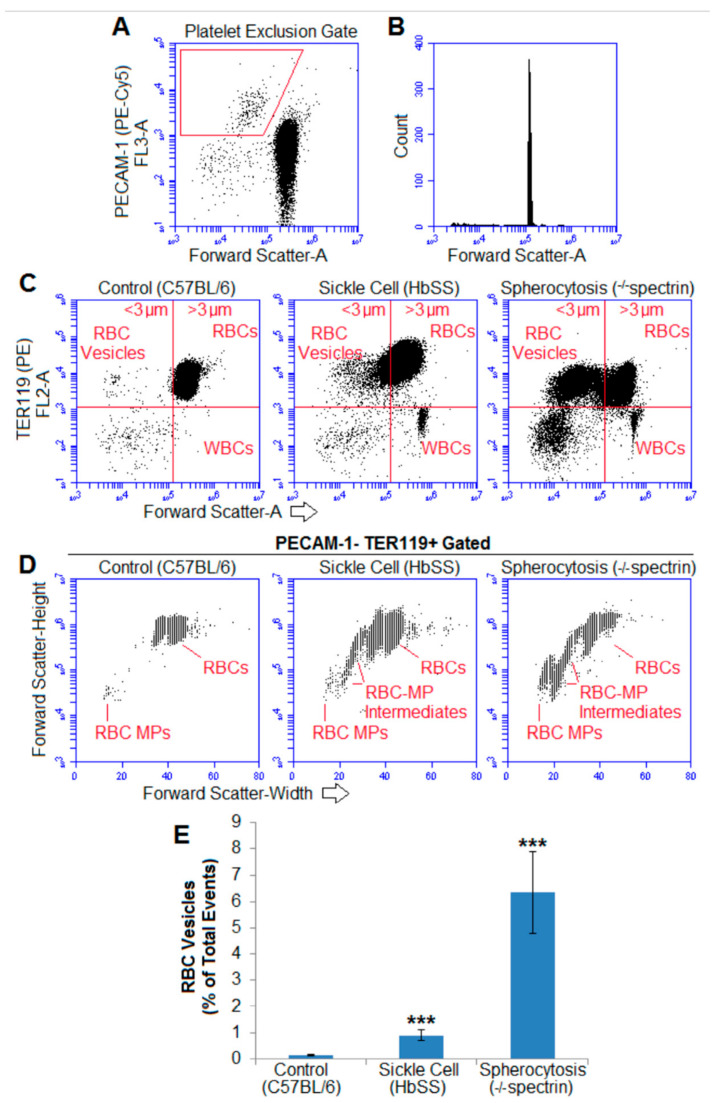
Blood from mouse models of hemolytic anemia contained large and heterogeneously sized vesicles: (**A**) PECAM-1+ platelets and other MPs/MVs were excluded (red trapezoid exclusion gate) and (**B**) 3 µm beads were used to distinguish vesicles from cells (as murine RBCs are smaller than human). (**C**) Representative (PECAM-1 platelet/MV excluded) whole blood diluted 1:1000 in normal saline with 10% BSA and stained for TER119 in healthy control mice (C57BL/6), mice expressing solely human sickle hemoglobin (HbSS), and mice with deficient spectrin modeling hereditary spherocytosis. (**D**) Representative forward scatter height by width demonstrates heterogeneously sized RBC-MV intermediates in both sickle cell and spherocytosis mouse blood. (**E**) RBC vesicles smaller than 3 µm made up nearly 1% of all events in the sickle cell mouse blood and over 6% of all events in spherocytosis mouse blood; *** *p* < 0.001 compared to normal mice; *n* = 3–5.

To ensure that the RBC vesicles were not actually RBC MVs adhered to or overlapping with platelets or platelet- or endothelial-derived MVs, PECAM-1 staining was employed to exclude such events (Figure 7A). Beads, 3 µm, were again used to qualitatively compare the sizes of cells and vesicles (Figure 7B).

The blood of both hemolytic mice groups showed subcellular RBC particles larger than 1 µm. Figure 7C,D is representative flow cytometry data of the (PECAM-1 excluded) blood of 3–5 mice per genotype. (The FSC-A of 3 µm beads is given for a size reference, and the fluorescence was based on isotype fluorescent controls). Less than 1% of total cytometry events from the healthy mice were TER119-positive and smaller than 3 µm (Figure 7E). However, significantly more heterogeneously sized RBC vesicles were found in the uncentrifuged blood of murine models of sickle cell disease and severe hereditary spherocytosis. These erythrocyte-derived particles had a wide size range based on their FSC (thus, size was not readily quantifiable).

## 3. Discussion

The majority of studies describing circulating MPs/MVs use centrifugation to remove cells and platelets before enumerating MPs/MVs. The results presented here directly challenge such practice. We showed that RBC-derived vesicles are not limited to less than 1 micrometer in diameter (a standard that is used for distinguishing platelets from platelet-derived MV) and suggest a spectrum of RBC-degradation product sizes. In addition, in congruence with one report directly enumerating RMVs in uncentrifuged blood [9], we showed that the concentration of RMVs decreases exponentially as centrifugation speed increases. Thus, when interpreting data comparing circulating MV concentrations in various diseases, the method to isolate and measure the MVs must be considered.

The principles of particle centrifugation can be described by the Stokes equation, in Equation (1):

(1) $$\nu =d^{2}*\left(p-L\right)*g/18n$$

where *v* is the sedimentation rate (velocity) of a spherical particle; *d*, the diameter; *p* is the particle, and *L* the medium densities; n is the viscosity of the medium, and, lastly, *g* is the gravitational force (RCF). Thus, for a given platelet MV compared to an identically sized RBC MV (assuming MV densities are the same as their respective parent cells, with RBCs nearly 1.1 g/mL (1.0996 g/mL) [48] and platelets only 1.069 g/mL [49]), RBC MV will sediment with a velocity 144% of that of the platelet MV.

Platelet-derived vesicles or MVs are reportedly the most abundant circulating MVs in the body, and as such, guidelines defining what an MV are largely aimed at excluding MVs from small platelets. Thus, platelet-derived MVs have been suggested as not being larger than 1 µm in diameter (particularly, between 0.5 and 0.9 µm) [50]. However, the data presented here represent a novel concept for what constitutes an erythrocyte-derived MP/MV, as well as their relative abundance. Typical small (<1 µm) platelet or cell-derived MVs are generated in response to activation or stimuli, including calcium- or ionophore-treated RBCs as a classic example. However, during hemolysis, there is uncontrolled destruction of erythrocytes, resulting in heterogeneously sized membrane-enveloped cell fragments (vesicles) as shown in this study. Previously MPs/MVs were considered inert cellular debris, but we now have widely available tools to examine these small and transient biological effectors. In like fashion, large RBC fragments have often been referred to as large “debris”; this paper challenges this distinction, suggesting that large debris and microparticles/microvesicles are differently sized vesicles on the same spectrum, resulting from damage and destruction of RBCs; and that the size upper limit of platelet MVs does not apply to RBC MVs.

The platelet MV size guideline has also been derived largely from studies of platelet MVs isolated from platelet-free plasma; however, this article demonstrates centrifugation preferentially removes large (RBC) vesicles. The sedimentation of any given particle depends on the centrifuge as well as properties of the particle and its solution [45]. Blood and plasma samples require an anticoagulant, and so when centrifuging biological fluids, the amount of liquid anticoagulant/saline needs to be considered. Otherwise, centrifugation of samples diluted in anticoagulant, additive solution, or saline (which are nearly half as viscous as plasma, primarily due to the lack of protein) will sediment vesicles faster than those with less anticoagulant. Thus, in order to study MVs in biological fluids and compare across institutions, further refinement of centrifugation as a means to isolate MVs is merited. Data presented here suggest centrifugation-free direct dilution/examination of the biological fluids should also be considered as a means to study MVs preferentially over centrifugation.

The population of cell-to-MV intermediate vesicles or “macroparticles” identified in this work is unlike other RBC vesicles identified before due to a number of reasons. Rather than use excessive heat, calcium, calcium ionophore or other artificial vesiculation agents, as has been reported elsewhere, we elected to focus on vesicles formed during hemolysis in more clinically or physiologically relevant scenarios. While RMVs derived in stored blood units may not be physiologic, they are still clinically relevant given such are transfused along with RBCs.

There are differences between RBC-bound hemoglobin and cell-free hemoglobin in their dynamics of gas exchange [21]. The biggest difference is the diffusion-limiting membrane, which causes intra-erythrocytic mM hemoglobin not to scavenge nitric oxide despite the favorable scavenging kinetics [51]. Additionally, principles of hydrodynamics result in a flow velocity-dependent RBC-free zone [52]. Thus, the physiologic half-life of endothelial produced NO is 1 ms rather than 1 µs as the case would be for (pathologic) cell-free hemoglobin. This is not necessarily the case for MV-containing Hb, as the RBC-free zone is penetrable by smaller vesicles that have been shown to be capable of gas exchange, namely, in scavenging nitric oxide [21]. Thus, there is likely no minimum size an RBC (or vesicular fragment thereof) can be to carry out the primary function of hemoglobin-mediated gas exchange. The size of a hemoglobin-containing vesicle, however, determines how close it will approach the vascular wall and whether or not it will scavenge endothelial-derived NO on the one hand or participate in tissue oxygenation on the other.

What is the minimum size a membrane bilayer-encapsulated hemoglobin-containing vesicle becomes an erythrocytic vesicle instead of an erythrocyte? Clinical diagnostic guidelines suggest that RBCs are microcytic if under 6–6.4 µm maximum in diameter, but the authors are unaware of a minimum size distinguishing a severely microcytic RBC from merely an RBC fragment as they transition along the size spectrum. A relevant article examining the ferric chloride-induced thrombosis model showed that RBC “fragments” with considerable heterogeneity in size played a substantial role in adhesion and thrombosis [30]. Thus, whether through scavenging of NO, seeding adhesion sites for thrombosis, or providing a phospholipid surface required for coagulation, RBC-derived vesicles are important pathological debris in hemolysis.

## 4. Materials and Methods

While flow cytometers were intended to count cells and not subcellular “debris” as vesicles have been called, flow cytometry is used extensively to evaluate microvesicles [34,43,44,50]. But as demonstrated previously, not all flow cytometer events are truly vesicles [36], so the findings on over two dozen expired units of RBC macroparticles in humans were confirmed with microscopy as detailed below and also demonstrated in mice models of human diseases. Lastly, as proof-of-principle, we showed that artificial RMVs sediment faster than platelet MVs and that fluorescent lipid-labeled RMVs can stick to RBCs as two ways to show how centrifugation can influence RMV enumeration.

### 4.1. RBC and RMV Isolation

Human blood: Outdated donor units are a known source of human RMVs [11,35]. While these may not be identical to physiologic RMVs, they are clinically relevant as they are transfused along with donor RBCs except in the rare instance a patient is transfusion dependent and needs washed cells [35]. Newly outdated leukocyte-reduced donor units (43–46 days after draw) were from the BloodCenter of Wisconsin (Milwaukee, WI, USA). Fresh blood was drawn (per Children’s Hospital of Wisconsin Institution Review Board protocol #CHW 91/34 GCRC 509, approved on 4/2/2014) from a healthy, consenting adult 9:1 into Acid Citrate Dextrose (ACD) and centrifuged at 500× *g* for 15 min at 22 °C to isolate RBCs and platelet-rich plasma. In some experiments, RBCs and platelet-rich plasma were subjected to extrusion through 0.45 µm pore size membranes to generate similarly sized MVs and, subsequently, centrifuged at different speeds before flow cytometry enumeration. Dilution of donor RBCs was into M199/0.2% BSA media with 0.1% EDTA in order to avoid glucose deprivation and subsequent hemolysis [53] or clotting.

Overnight sedimentation was performed by gently mixing and then sterilely withdrawing 10 mL of blood from the outdated unit bag through a 16× *g* needle to avoid any mechanical lysis, and then slowly transferring that 10 mL to centrifuge tubes that were then allowed to sit upright overnight for 12–16 h. To prevent any change in temperature, all this was done in a 4 °C room.

Mice: Characteristics of MVs in healthy human volunteers have been shown to vary by gender and menstrual cycle [54], diet [55,56], smoking status [56], and race [57]. To bypass such heterogeneities, well-characterized mouse models of human hemolytic anemia were examined. Berkeley Sickle Cell Disease (SCD) mice have a phenotype with features of severe human SCD [58,59]. The WBB6F1-sph/sph mice have severe hereditary spherocytosis due to the fact of a deletion in the erythroid α-spectrin gene [60]. Mice protocols were approved by the Institutional Animal Care and Use Committee of the Medical College of Wisconsin, protocol #AUA00000142 approved on 30 January 2014. Blood was collected from anesthetized mice by cardiac puncture using 20 gauge needles and drawn 5:1 into ACD and diluted 1:1000 in sterile–filtered normal saline with 10% BSA. (This saline is slightly hypotonic for murine blood but was used to avoid further dehydration and possible subsequent spiculation/vesiculation [53].)

### 4.2. Cell and MV Staining

Incubations were done on ice at 4 °C in the dark for >15 min unless otherwise stated. Anti-human glycophorin A and anti-murine TER119 antibodies were from eBioscience (San Diego, CA, USA) and added to cell suspensions at 1:500–1:1000. Glycophorin A, also known as CD235a, is uniquely exposed on the surface of red blood cells and is associated with the RBC lipid bilayer [61,62]. This protein is widely accepted by hematologists as a marker specific for RBC membranes and was chosen as a way to confirm the origin of measured particulate was from RBCs. TER119 is a molecule associated with murine glycophorin A, and is used similarly as a marker of RBCs [47]. Anti-PECAM-1 antibodies (from Becton Dickinson, Franklin Lakes, NJ, USA) was used as a marker of platelet or endothelial origin, and staining was performed with anti-PECAM-1 antibodies as reported previously [11,36] to excluded such events for subsequent flow cytometry experiments.

Lactadherin-FITC (Haematologic Technologies Inc., Essex Junction, VT, USA) was used as a calcium-independent PS-probe as per the manufacturer’s instruction (using 1:100 final dilution). Fluorescent lipid-labeling of MVs was done by incubating MV-rich supernatant (isolated by centrifugation at 1500× *g* for 15 min at 4 °C) with 20 µg/mL DiI (a highly-lipophilic membrane dye) at 37 °C for 20–30 min. The MVs were then isolated by centrifugation at 15,000× *g* for 15–30 min and washed with M199/2% BSA with 0.1% EDTA. The RBCs were resuspended in the third wash buffer to control for residual dye. The DiI-MVs were incubated with cells for 15 min at 37 °C.

### 4.3. Flow Cytometry

Flow cytometry was performed on an Accuri C6 (BDIS, San Jose, CA, USA) as described previously [10,41]. The Accuri C6 forward scatter height (FSC-H) threshold was 10,000 as this provided an acceptable minimum background of less than 100 events per minute on sterile–filtered saline. Beads with various diameters were used to estimate vesicle size and confirm the cytometer reported concentrations as described elsewhere [10,22]. Samples were gently mixed immediately prior to flow cytometry. Analysis of data was conducted using the C-Flow Plus (BDIS). The linear range of the cytometer was established by diluting 3–4 samples in saline and comparing the event rate; the linear range was determined by minimizing the variance from a linear fit of the data through 3–5 points.

### 4.4. Fluorescence Microscopy

Fluorescently labeled cells and RMVs were plated and immediately imaged on a Nikon TE200 (Melville, NY, USA) using 100× magnification. Images acquisition was via Photometrics CoolSNAP-ES camera (Tucson, AZ, USA) using Metamorph (Molecular Devices, Sunnyvale, CA, USA). Image brightness and contrast were adjusted using ImageJ (US NIH, http://imagej.nih.gov/ij/).

### 4.5. Statistical Analysis

Statistical analysis was conducted using the Student’s 2-tailed *t*-test without assuming equal variance. One-way ANOVA was used to compare multiple manipulations. For data that varied by an order of magnitude, data were first log-transformed prior to applying the *t*-test. An alpha of 0.05 determined significance. Data are presented as the mean ± standard error of the mean.

## Figures and Tables

**Table 1:** Various sizes of red blood cell (RBC) vesicles including the method to isolate them from the parent cell. * Gating excluded events that were larger than 1 µm to rule out platelets.

Source of RBC Vesicles	Cell/Vesicle Separation	Vesicle Diameter	Examination Method(s)	Reference
(Patho)physiologic RBC Damage or Hemolysis				
Malaria	1500× *g* for 15′	≤1 µm *	Flow cytometry	[37]
Hemolytic anemia, ±overnight storage	1500× *g* for 5’ to remove cells	~0.25–0.30 µm	Light microscopy	[8]
Autoimmune thrombocytopenia	200× *g* for 8’ to remove cells	Up to 2 µm	Electron microscopy	[5]
Various diseases	1550× *g* for 20’ to remove cell	0.05–1 µm	Electron microscopy	[38]
During blood storage	≥870× *g* for 20’ to remove cells, direct dilution	3 µm, 2.78 µm	Light microscopy, flow cytometry	This study
Laboratory Techniques				
Heating RBCs to 45 °C, 42 °C with pH ≤ 6	Filtered through 3 µm membrane	1 up to 3.5 µm	Electron microscopy	[39]
Heating RBCs to 45 °C with EDTA and Ca^2+^	Sucrose gradient centrifugation	0.5–1 µm	Light and electron microscopy	[40]
Ionophore A23187 and Ca^2+^	Centrifugation to sediment cells	60 nm, 100–150 nm	Electron microscopy	[41,42]

## Data Availability

Data are available upon reasonable request to the corresponding author.

## Abbreviations

FSC	Forward Scatter
MP/MV	Microparticles or microvesicles
PECAM-1	Platelet Endothelial Cell Adhesion Molecule-1
PMV	Platelet-derived microvesicles
RMV	Red blood cell-derived microvesicles
SSC	Side Scatter
PS	Phosphatidylserine
SCD	Sickle Cell Disease
